# Big food and drink sponsorship of conferences and speakers: a case study of one multinational company’s influence over knowledge dissemination and professional engagement

**DOI:** 10.1017/S1368980022002506

**Published:** 2023-05

**Authors:** Jónas Atli Gunnarsson, Gary Ruskin, David Stuckler, Sarah Steele

**Affiliations:** 1 Department of Economics, Bocconi University, Milan, Italy; 2 U.S. Right to Know, Oakland, California, USA; 3 Department of Social and Political Sciences, Bocconi University, Milan, Italy; 4 Intellectual Forum, Jesus College, Cambridge, UK; 5 Cambridge Public Health, University of Cambridge, Cambridge, UK.

**Keywords:** Industry influence, Public health event, Conflict of interest

## Abstract

**Objectives:**

Research identifies that multinational corporations, including The Coca-Cola Company (‘Coca-Cola’), seek to influence public health research and policy through scientific events, such as academic and professional conferences. This study aims to understand how different forms of funding and sponsorship impact the relationship between Coca-Cola, academic institutions, public health organisations, academics and researchers.

**Design:**

The study was conducted using Freedom of Information (FOI) requests and systematic website searches.

**Setting:**

Data were collected by twenty-two FOI requests to institutions in the USA and UK, resulting in the disclosure of 11 488 pages, including emails and attachments relating to 239 events between 2009 and 2018. We used the Wayback Machine to review historical website data to evaluate evidence from 151 available official conference websites.

**Participants:**

N/A

**Results:**

Documents suggest that Coca-Cola provides direct financial support to institutions and organisations hosting events in exchange for benefits, including influence over proceedings. Coca-Cola also provided direct financial support to speakers and researchers, sometimes conditional on media interviews. Also, indirect financial support passed through Coca-Cola-financed non-profits. Often, such financial support was not readily identifiable, and third-party involvement further concealed Coca-Cola funding.

**Conclusion:**

Coca-Cola exerts direct influence on academic institutions and organisations that convene major public health conferences and events. These events offer Coca-Cola a vehicle for its messaging and amplifying viewpoints favourable to Coca-Cola’s interests. Such corporate-sponsored events should be viewed as instruments of industry marketing. Stronger rules and safeguards are needed to prevent hidden industry influence, such as complete disclosure of all corporate contributions for public health conferences and their speakers.

Academic and professional conferences are important events in public health and nutrition calendars, offering participants the opportunity to interact and to develop the future agenda in the field and beyond, while allowing policymakers and practitioners to obtain up-to-date evidence that informs practice^(1)^. Consequently, these events directly influence research, collaboration, policy and practice. With the rapid return of these events in the post-COVID-19 calendars, many events are now blended, offering online and in-person attendance, expanding reach and influence, and thus demanding evolving best practice in managing conflicts of interest.

Evidence from a broad range of fields suggests commercial entities seek to leverage events, specifically to promote information, viewpoints and scholars favourable to their commercial interests^(2)^, and to promote their products^(3)^. Past studies have shown they deploy ‘opinion-leaders’ – those able to influence their peers – paying up to $3000 for a single lecture at a conference, with talking points provided by the company^(4)^. Studies also show companies find value in accompanying media and press releases seeing them as endorsements^(5)^, which give the impression that a position or product is promoted by an ‘expert source’ more widely^(6)^. Studies of the tobacco industry identified instances where it sought to shape the narrative through conferences or, where it could not, engaged in media strategies to deflect attention from the public health messages these events contained^(7,8)^. Research on the pharmaceutical industry identified billions spent by companies to interact with health professionals^(9)^. Other research identifies that some companies, or the organisations they fund, hold conferences at prestigious institutions, with accompanying publicity using the institution’s name prominently, to imply endorsement, even though facility rental was the only connection^(10)^.

An emerging body of evidence reveals that the food and beverage industries employ similar tactics^(11–13)^, buying exhibits and advertising in conference materials, while underwriting food and beverage provision, providing gifts and giveaways, and offer awards and travel funding. This sponsorship not only influences what is presented and discussed, but what is not, with one Brazilian conference cancelling a debate on childhood obesity because of fears that it would deter sponsors^(14)^. However, researchers note that industry influence at public health and nutrition conferences is often not visible^(15)^.

Here, we investigate the potential influence and intent to influence of The Coca-Cola Company (hereafter, ‘Coca-Cola) on public health institutions and organisations, associated conferences and events, and the researchers involved. We systematically evaluate a set of documents obtained through Freedom of Information (FOI) laws, in concert with Internet Archive materials, to analyse Coca-Cola’s involvement, and how the company works with the organisers and sponsored researchers.

## Methods

Between July 2015 and 2018, US Right to Know (USRTK), a non-profit investigative public health group, made a series of FOI requests under state laws to public institutions in the USA, and under the Freedom of Information Act 2000 (FOI) to public institutions in the UK. The institutions were selected because their researchers either had acknowledged the receipt of corporate funding or were known to work with industry in their research. Subsequently, USRTK received twenty-two responses containing a total of 11 488 documents, including email threads and attachments from January 2012 to July 2018. The FOI document requests are listed in Appendix I.

The documents were initially reviewed by GR or his colleague Rebecca Morrison. Three other researchers then read the documents independently and manually, and JAG read and identified mentions of events in which public health researchers were invited to as academics and Coca-Cola was financially linked to, according to the information that was available in the emails and attachments. Coca-Cola’s influence was classified into three categories: (a) direct financial support to a conference or its organisers; (b) financing of non-profit bodies, which then sponsored events; or (c) financial support to the speakers, panellists and researchers for attending conferences. This categorisation was independently checked and validated by DS.

To validate the status of the sponsorship, and to check whether financial support from Coca-Cola was disclosed and reduce interpretive bias, we reviewed websites of the conferences identified, supplemented with searches of the Wayback Machine – an archive of websites which stores snapshots for future reference^(16)^. We also used the website to determine whether the conference or event page overtly mentioned sponsorship, either of the whole event or part of it, by Coca-Cola.

## Results

We identified 239 events, both public and private, in which the researchers mentioned in the FOI emails were invited to as academics, listed in Appendix II, with Coca-Cola funding 158 of them, either directly or indirectly. We found evidence that Coca-Cola funded ninety-eight conferences, twenty-one symposia, ten lecture rounds, fourteen private meetings, one workshop, three webinars, three seminars, three forums and three panel discussions.

Of the 158 events in our dataset supported by Coca-Cola, thirty-eight were related to obesity in general, thirty-four to physical activity, twenty-one to nutrition, twelve to collaboration between academia and private companies, nine to the use of sweeteners, six to paediatrics, five to Coca-Cola itself, three to diabetes and one on a soda consumption tax (see Appendix II).

Of the 158 events identified, twenty-eight involved Coca-Cola contributing funds directly to the organiser, seventy were funded by third parties that received funds from Coca-Cola and sixty involved Coca-Cola funding speakers directly. In nine cases, we found evidence that Coca-Cola supported both the conference organiser and the speakers directly.

In the twenty-eight cases where Coca-Cola contributed funds to conference organisers between 2009 and 2016, payments ranged from $2500 to $100 000 per event (Appendix III). However, our data on the range of funding is limited, as it is only based on six observations. Coca-Cola disclosed its support to many of these events on a special ‘Transparency List’ on the Company’s website in 2018. Coca-Cola has now removed the list from its website, but it can still be accessed by using the Wayback Machine^(17)^. In many cases, Coca-Cola was an official sponsor and donated directly to the host organisation. For example, Coca-Cola agreed to be the keynote sponsor for the National Physical Activity Plan Congress in 2015, with a $30 000 payment, which allowed the company to influence the conference agenda by selecting a keynote speaker, as well as to advertise at the conference venue^(18)^.

Coca-Cola paid a more active role in some conferences by being an official sponsor in addition to paying individual researchers to participate. This was the case for the International Convention on Science, Education and Medicine (ICSEMIS) in Glasgow in 2012, where the Company was listed as the main sponsor on the conference website^(19)^. One email from a Coca-Cola representative explained to Dr Steven Blair, a professor in public health at the University of South Carolina, Coca-Cola’s plans to host a session at ICSEMIS:

*[…] and myself have had a recent conference call to discuss the possibility of Coca Cola hosting a 2-hour Master Class at the ICSEMIS conference to teach physicians (GPs) how to motivate patients to be physically active as part of their health care.* ^(18)^

Coca-Cola also invited Blair to speak at this conference. In another email about ICSEMIS, Dr Rhona Applebaum, then Coca-Cola Chief Health and Science Officer, invites Blair to speak at one of Coca-Cola’s organised sessions, adding that:

*Of course we will cover all expenses.* ^(18)^

In a following email, Blair accepted this offer. The same thing occurred when Coca-Cola was an official sponsor of the 2014 Nutritional Society of Australia’s Annual Conference and the European College of Sport Sciences Annual Congress the same year^(20,21)^. We found evidence that it funded both the event itself and expenses and honoraria for selected participants.

In our emails, Coca-Cola also acknowledges how important it is to amplify specific viewpoints among public health scientists, while it also admits using key opinion leaders to do amplify these viewpoints. After having accepted to speak at the Coca-Cola-funded 10th International Symposium on Body Composition, Dr Diana Thomas, a professor at Montclair State University, receives the following email from a Coca-Cola employee:

*Many thanks for your collaboration. It is very important for us to amplify your scientific and health messages as Health Key Opinion Leaders.* ^(18)^

We note that in nine out of the fifty-eight events where Coca-Cola funded a speaker, Coca-Cola requests in their invitations for them to talk to the media^(22)^. For example, Coca-Cola offered to pay $18 000 in travel expenses for a research team to participate in a symposium in South Africa in 2013^(18)^. An email from a Coca-Cola employee to the head of the research team stated:

*In terms of your visit to SA, we would like to ask if you and the ISCOLE team: […] would consider participating in media interviews/discussions regarding your research and also topics related to it.* ^(22)^

In another email sent a year earlier, Dr Applebaum sends the head of the same research group the following regarding an upcoming conference in Spain:

*Do you want to speak to the media on Iscole?* ^(22)^

Coca-Cola paid the research team $15 000 in travel expenses for that conference^(18)^.

A similar request followed in an invitation from Coca-Cola to Dr Hand to participate in a conference hosted by the South African Sports Medicine Association in 2015^(22,23)^. An email from Coca-Cola to Dr Hand includes the following:

*We would also like to ask if possible, if you might be amenable to meeting with other stakeholders in the field of public health in SA and potentially also the media?* ^(22)^

This practice, found in several other cases, seems to be a part of a Coca-Cola strategy to promote the messaging of scientists whose views align with Coca-Cola interests^(22)^.

Additionally, funding gave Coca-Cola opportunities to influence a programme. For the International Conference on Physical Activity and Public Health (ICPAPH) in 2012, a donation of $100 000 gave Coca-Cola opportunities to shape the conference schedule^(18,24,25)^. An email from Dr Applebaum sent to a group of public health scientists states:

*As you know – our sponsorships of ICPAPH allowed us two panels—which we had no part in designing other than suggesting a topic area.* ^(18)^

According to the programme, these topics were on sedentary behaviour and the future of physical activity policy and research^(26)^.

Coca-Cola also supported sixty conferences indirectly, by funding other organisations. Although most of these organisations disclose their affiliation with Coca-Cola, the scale and nature of their relationship with the Company was not always clear. For example, the main sponsor of the ICPAPH in 2012 was the Beverage Institute for Health and Wellness, donating $100 000 (Appendix III). Coca-Cola set up this body in 2004 ‘to use evidence-based science to advance knowledge and understanding of beverages, beverage ingredients, and the important role that active healthy lifestyles play in supporting health and wellbeing’ and was headed at that time by Dr Rhona Applebaum, while she was still working for Coca-Cola^(27)^.

We found several other instances of indirect funding to academic events through Coca-Cola-affiliated groups. One of these groups is called Exercise is Medicine (EIM), which was founded and funded by Coca-Cola but managed by the American College of Sports Medicine. Coca-Cola gave $968 000 to fund EIM between 2010 and 2015^(17)^. Despite being ostensibly independent of the Company, our emails show that Coca-Cola could influence whether EIM was present at scientific conferences, as became evident in the World Congress of Cardiology Science in 2012, where Applebaum suggests an exhibition booth for EIM and offers support from Coca-Cola. In an email answering Dr Carl Lavie, a professor of cardiology at the University of Queensland, she responds to him tells her that EIM will host a lecture at the conference with:

*This is great, I’m thinking EIM needs to have a presence here—somewhere Chip can direct folks to for more info. I’m thinking a booth in the exhibit area. Views? We can try to help here.* ^(18)^

Lavie responded positively to this suggestion in a following email. The close relationship between Coca-Cola and EIM was apparent in many ways. In addition to obtaining Coca-Cola’s help in arranging exhibition booths at academic events, EIM hosted international conferences in close collaboration with Coca-Cola and its initiatives. An example is the EIM World Congress in 2015, where Dr James Hill, a researcher at the University of Alabama, Birmingham, was invited as a keynote speaker by Dr Steven Blair, who was on the EIM advisory board at the time^(28)^. In an email to Hill, Blair writes:

*Jim Skinner and I would like for you to give the keynote lecture for the Exercise Is Medicine World Congress, and the topic would be ‘The Global Energy Balance Network’.* ^(18)^

Hill accepts this invitation in a following email. The Global Energy Balance Network (GEBN) was a Coca-Cola initiative, aimed at convincing public health professionals to emphasise physical inactivity, instead of sugary beverages, as a major cause of obesity^(29,30)^. Both Hill and Blair were leaders of GEBN, which presented at multiple conferences hosted by EIM. In addition to the Network’s presence at the EIM World Congress in 2015, we found that Blair was invited as a speaker at another EIM conference hosted by the Royal Society of Medicine in London the same year. In an email answering the invitation, Blair writes:

*Seriously, I will plan on focusing on energy balance and our Network. I also have new data from our Energy Balance Study.* ^(18)^

Just as with the GEBN, the Energy Balance Study (EBS) focused on physical inactivity as a major cause of obesity and was primarily funded by Coca-Cola. The Company disclosed its funding towards both initiatives on a list of its financial contributions on its website in 2018, showing a donation of $4·4 million to the EBS and $1 million to the GEBN. The list is no longer accessible on the Company’s website, but it can be accessed by using the Wayback Machine^(17)^.

Notably, Coca-Cola has relationships with other non-profit organisations including the International Life Sciences Institute (ILSI), which was founded by former Coca-Cola Senior Vice President Alex Malaspina, and Coca-Cola also has funded ILSI. Previous research shows that ILSI has been an important vehicle for Coca-Cola to exert influence around the world, on policy, researchers, events, policymakers and practitioners, taking a role in promoting messaging that can further commercial interests^(31–35)^. ILSI supported thirty of the events we identified. Sometimes, ILSI offered to act as a conduit to reimburse public health scientists through Coca-Cola by having them deliver presentations with selected topics. This was the case at the ILSI South Africa Risk Assessment Conference in 2014, where an ILSI employee wrote to a researcher at Imperial College London:

*Hope all’s well with you? Are you free to speak at the above meeting, subject to us getting Coke to pay ? Topic would be ‘Food Additives and Risk Assessment’.* ^(18)^

However, the researcher turns down this offer in a following email. Coca-Cola invited researchers through ILSI at private stakeholder events as well. For example, ILSI invited Dr Russell Pate, a professor of public health at the University of South Carolina and then-Chair of the National Physical Activity Plan, which Coca-Cola has funded and is now a part of the Physical Activity Alliance, to attend a private forum in Colombia in 2014 stating^(17,36)^:

*Dear Dr. Pate, My name is Olga Mora, Executive Director of the North-Andean International Life Science Institute, ILSI. As Alexandra Echeverria (Coca Cola Team) wrote you on Tuesday, July 29, we want to invite you as one of the academic speakers in a forum that will be held on September 4th, in Bogotá-Colombia….* ^(18)^

Pate accepts this invitation in a following email. In other emails, ILSI employees explain that the forum was hosted by representatives of the private sector in Colombia and would be about the harms of a soda consumption tax^(18)^.

The emails reveal Coca-Cola was also a founding member and an official sponsor of the EPODE International Network, an organisation supposedly aimed at tackling childhood obesity^(37)^. EPODE hosted the Global Obesity Forum in 2012 and, according to our emails, Coca-Cola paid Dr Steven Blair $1877 to deliver his presentation^(18)^.

Another example of Coca-Cola paying for a researcher through another organisation is the company’s endorsement for a researcher at Imperial College London who participated in a private meeting by the European Scientific Advisory Council (ESAC) in 2013. ESAC is a federation of European policymakers. Our emails indicate that the meeting was about low-calorie sweeteners. Coca-Cola offered to cover all travel expenses for the researcher as well as paying a participation fee through another agency, as can be seen in the following email from a Company employee:

*Dear [BLANK], I didn’t have the pleasure of formally meeting you during the ESAC meeting but I am working with [BLANK] and [BLANK] on the low-calorie sweeteners project for The Coca-Cola Company. I’m reaching out to you with regard to the fees and expenses related to your participation in ESAC – wanted to let you know that payment for these will be made through our PR Agency…* ^(18)^

In addition to sponsoring organisations that were founded by Coca-Cola employees, Coca-Cola also often worked as a contact – an intermediary – between various public health institutions and scientists attending conferences. For example, the documents show Coca-Cola’s close collaboration with the American Academy of Pediatrics (AAP), the Academy of Nutrition and Dietetics (AND), The Institute for Excellence in Pediatrics (INEiP), The Obesity Society (TOS) and the American Academy of Family Physicians (AAFP). In one email, Applebaum writes to Dr Peter Katzmarzyk and Dr Timothy Church, professors at Louisiana State University, stating that she will try to arrange for them to give them a presentation at the AAP National Conference in 2014. Following up on that email, Applebaum writes:

*As you know AAP is a great partner of ours…* ^(18)^

We could not, however, find mention of them in the conference programme, and therefore cannot be sure if they gave or did not give a presentation.

Similarly, we found evidence of Coca-Cola acting as an intermediary for AND by inviting a scientist to an academic event. In 2012, Joan Koelemay, the director of Health and Wellness programmes for Coca-Cola, writes the following email to Dr Joanne Slavin, a professor at the University of Minnesota:

*Hi Dr. Slavin! By way of introduction, I am the director of Health & Wellness Programs for Coca-Cola […] I manage the professional meetings related to our health professional partnerships, including our support of the Academy of Nutrition and Dietetics – which is why I’m contacting you! We would like to invite you to speak at the Academy of Nutrition and Dietetic […] Nutrition News Forecast symposium…* ^(18)^

Slavin responds positively to this invitation in a following email. At the Nutrition News Forecast symposium, which was organised by the AND, Coca-Cola invited Slavin to speak at a Company-sponsored scientific event and put the media focus on the dangers of sugars in the diet ‘into [scientific] context’ ^(18)^.

Also, Coca-Cola invited Dr Gregory Hand, founding dean of the West Virginia University School of Public Health, to the same symposium in 2015, asking him to speak about GEBN^(38)^. Emails show that Coca-Cola acts as a go-between with Hand and AND, receiving Hand’s presentation slides and forwarding them to the institution^(18)^. In one email, Koelemay writes to Hand:

*Good morning! So sorry to be a pest, but we do need to get a copy of Dr. Hand’s slides and his bio to AND in the next day or so. Please???* ^(18)^

Coca-Cola reimbursed Hand for all of his expenses as well as providing him with a $1500 honorarium for presenting at that event^(18)^.

Other emails also revealed the close partnership between Coca-Cola and AAFP. In one of them, L. Celeste Bottorff, then the Company’s Vice President of Health and Wellbeing Initiatives, wrote that she had:

*A great relationship with the AAFP.* ^(18)^

Moreover, we also found that Coca-Cola had acted as an intermediary between the AAFP and public health researchers. At the AAFP Nutrition Theater in 2014, a Coca-Cola employee is a contact between Dr Hand, who was invited to speak at the event, and the AAFP. In an email to Hand shortly before the event, the Coca-Cola employee writes the following:

*Hi Dr. Hand, As we are getting close to AAFP I wanted to share the final poster for your nutrition theater session. […] Finally, if at all possible, if you wouldn’t mind – can you share your presentation prior to the session?* ^(18)^

Additionally, we found two cases of public health organisations asking Coca-Cola to sponsor their conferences. In 2015, Dr Diana Thomas, a member of TOS, asks Susan Roberts, Director of Health and Nutrition at Coca-Cola, if it is able to sponsor a scientific session at their annual meeting^(18)^:

*I hate to talk to you regarding funding, but I’m the president of the Basic Science Section of TOS and they asked us to reach out to industry to ask for funding for the TOS BSS meeting next fall.* ^(18)^

Similarly, Russel Hale, a member of INEiP, sends Applebaum at Coca-Cola an email in 2015, asking for company funding^(18)^:

*I wanted to make contact early on this year to avoid the rush we had in 2014 to make sure the funding, speakers and even details such as the correct hotel used for Coca-Cola participants are put in place way ahead of time.* ^(18)^

In both these instances, Coca-Cola agreed to support the organisations by covering expenses for selected researchers at their conferences.

Additionally, we found sixty scientific events in which Coca-Cola-supported conference speakers by directly funding their participation, including through speaker fees and travel costs. We could not identify disclosure of this support in the conference programmes through internet searches. In nine out of those sixty conferences, Coca-Cola employees also asked the sponsored researchers to talk about their findings in media interviews^(22)^. Most Coca-Cola speaker support related to two major studies on the causes of obesity: The International Study on Childhood Obesity, Lifestyle and the Environment (ISCOLE) and the Energy Balance Study (EBS)^(22)^. Coca-Cola funded both studies, which focused on physical inactivity as a major cause of obesity.

Many researcher support arrangements were in the form of an honorarium. For example, Coca-Cola paid a public health researcher at Imperial College London for participating in the Panhellenic Diabetes Congress in Greece in 2013. According to an email from a Coca-Cola employee to the researcher, Coca-Cola was willing to pay for his presentation:

*Apart from your honorarium (3000 euros) we have estimated an additional amount of 300 euros for transportation and other miscellaneous costs you may have had.* ^(18)^

At another conference in Greece, the 8th Diets Conference, organised by the European Federation of the Associations of Dieticians (EFAD) in 2014, Coca-Cola paid Hill to participate in a symposium by EFAD, by covering both his travel costs and accommodation, in addition to inviting Hill and his wife out for meals^(18)^. In another email, the employee promised Hill not just full support for travel expenses, but also an honorarium and invitation to a dinner with Coca-Cola’s CEO:

*Jim hello, Apologies for the delay! We are exchanging emails with Tim about the flight bookings and we will organise everything. Business class of course. The honoraria is fine and ISA will cover this entirely. […] Since the honoraria covers ISA but also some of the Coke activities, Coke will take care of all flights and accommodation costs. In terms of meeting with James Quincy, our CEO, and his leadership team, it seems that this will take place on Tuesday 14th, over a lovely dinner.* ^(18)^

The honorarium, which was $5000 according to another email, came from the International Sweeteners Association, a lobby group linked to Coca-Cola^(18)^. We were unable to identify acceptance and transfer of funds in the emails received.

According to an email from Applebaum, the Company also provides different types of funding to researchers at public universities than those at private institutions. Before a 2015 conference hosted by the Chinese government and Coca-Cola, Applebaum reaches out to Dr Timothy Church, a professor of preventive medicine at Louisiana State University, to see if he could give a presentation there^(39)^. When Church asks for an honorarium, Applebaum answers:

*Depends—what are you? Private or Public? If private we can (but the bank is not very healthy; if public (e.g. LSU) will provide an unrestricted grant to your dept.* ^(18)^

The differences between Coca-Cola’s funding of public and private university scientists suggests that our set of emails may not be representative of Coca-Cola’s practices, as FOI legislation largely only applies to researchers at public universities in the UK and the USA.

Coca-Cola also invited scientists to closed forums for stakeholders to present results from company-funded research projects and discuss how to promote their scientific agenda. An example is the ‘Balancing the Debate’ symposium, which the Company hosted in its headquarters in Atlanta in 2012^(18)^. According the invitation that Coca-Cola sent to a selected group of public health researchers, the theme of the symposium was ‘Coca-Cola’s Support of Evidence-Based Science, Systematic Reviews & Science Advocacy’. An invitation sent to Dr Gregory Hand, a professor of public health at West Virginia University, titled^(38)^: *‘CONFIDENTIAL: Energy Balance – Internal Workshop. The Coca-Cola Company’*, contained information on how the workshop would help promote a favourable narrative in public health, called Energy Balance:

*We are planning to organize in Coca-Cola (Brussels), an internal session about Energy Balance […] We would like to know if you may be interested in to participate in this Workshop […] the workshop will provide an opportunity to increase the Coca-Cola EB strategy in: 1. Science […] 2. Business […] 3. Policy makers and Public Health…* ^(18)^

Notably, despite the emphasis that Coca-Cola places on media coverage of company-funded projects, we found one instance in our document set where Coca-Cola did not want to be associated with them. In 2012, Coca-Cola invited Dr Steven Blair to give a presentation on physical activity at a conference in Turkey. Although Coca-Cola wanted Blair to participate in a press conference after his talk, Isil Ulger, Coca-Cola’s Scientific Manager in Central Asia, explicitly stated that there would be no mention of Coca-Cola sponsorship at the event, according to one of our emails:

*After our session we are trying to have a (media)press meeting with some members of Turkish health press. (Coke name, sponsorship will not be mentioned for sure!)* ^(18)^

Thus, by funding Blair, Coca-Cola was able to place a speaker at a conference to talk about a topic of their own choice and have his message conveyed by local media, without it being seen as a sponsored message. This was a notable exception.

## Discussion

We found evidence of all three types of support in the documents: (1) funding conference organisers, (2) funding through non-profit organisations and (3) funding conference speakers privately. These different forms of support could result in numerous benefits for Coca-Cola. Coca-Cola often obtained advantages in exchange for funding like the opportunity to propose topics for keynotes, to suggest or place plenary speakers, and to undertake marketing. Other funding enabled lunchtime seating with conference VIPs and advertisement of their brand at the venue^(18,40)^. The effect of this industry involvement is to expose professionals to the brands and marketing of certain products, including ultra-processed foods and sugar-sweetened beverages, while also allowing the brands to build their image by affiliating with scientific and research communities^(41)^.

Where individuals lack knowledge about industry influence and conflicts of interest, events may therefore become a mechanism for industry to access and tacitly influence researchers, even hiring them, something particularly relevant to consider around early career individuals^(42)^. The instances of funding through third parties suggest there may be researchers who find themselves in the situation of taking support and then, only subsequently, realising who they have taken it from, after a company attempts to or inputs into studies or other activities, or when introductions to prominent individuals from the corporate entity take place^(35)^. Notably, this influence and the benefits for industry flow long after conferences themselves impacting on research agendas and collaborations, and with funding generating goodwill among researchers towards the company^(43)^. It is critical to research and understand such engagement of public health experts to amplify corporate messaging because credible and trusted experts may appear as neutral and informed and thereby influence policymakers, practitioners and the public at large, with medical doctors being especially trusted by the public^(44–47)^. The failure to declare conflicts of interest and funding sources obscures corporate influence over what is said and to whom it is stated.

Furthermore, even if researchers are not influenced in practice, the involvement of the industry in these events risk the public and peers perceiving influence, potentially impacting trust and credibility of both of the research and the researcher’s professionalism and expertise^(42)^. Education on conflicts of interest, and how to avoid them, is especially important considering the use of third parties funded by industry as conduits for travel funding. Also, with increasing pressure to find funding, particularly private support, placed on researchers and academics across the higher education sector in most countries, training on conflicts of interest is critical at the earliest stages of research careers.

Also, we are concerned about the linking of funding to the undertaking of media coverage. By pushing speakers towards the media, a company’s influence over science communication may be significant and therefore should be fully disclosed^(2)^. With online coverage not a common feature of conference, webinar content and press even has the capacity to spread worldwide, being present for many years on sites like YouTube. Concealed influence therefore matters as Coca-Cola has clear interests in spreading favourable conference content internationally, including content which runs counter to public health^(48)^.

## Conclusion

Coca-Cola has funded numerous professional conferences, events, activities, and stakeholder talks, both overtly and covertly. The documents we obtained show that Coca-Cola seeks to place favourable speakers and influence conference panels through direct funding and via third-party conduits. Public health organisations have sometimes abetted this process, often receiving financial support, either from Coca-Cola or Coca-Cola-funded organisations, for hosting conferences and other academic events. In so doing, the Coca-Cola may be able to influence conference agendas, including by choosing speakers, workshops and plenary speakers. This evidence corroborates other evidence that Coca-Cola attempts to cultivate relationship with leading public health organisations^(49,50)^.

### Study limitations

Before interpreting our findings further, we must note several important limitations. First, our study did not observe actual downstream impacts on population or whether such attempts to influence conference agendas succeeded in doing so. Second, as with all FOI studies, our document set is partial. It is impossible to ascertain whether FOI responses form a complete sample of communications between Coca-Cola and associated researchers. There is always a possibility that some relevant documents were not provided and/or withheld. Certain documents may be excluded in accordance with provisions in relevant legislation, and the research team may not have known these documents were redacted or removed prior to the relevant body providing the data in response to the request.

Second, it is impossible to capture the full universe of Coca-Cola-funded conferences, as we only detect those referenced in FOI-released email exchanges. Ideally, Coca-Cola would fully disclose conference activity. Yet as we found, often corporate funding is not disclosed in conference programmes.

Third, while we sought to retrace online records of historical conferences, the Wayback Machine was unable to retrieve information from all the academic events identified in the dataset. The Wayback Machine captures snapshot data on websites on a fixed date or set of dates, rather than indexing content and allowing for notation of changes. It is possible, therefore, that a website was updated after the indexation to note the sponsorship.

Fourth, as with all mixed-method research, there is the potential for bias in interpreting qualitative data. We report extracts as they appear in the agreements and quote any related emails ‘in their own words’ to allow readers to critically assess our interpretations. Also, members of the research team engaged reflexively and discussed all interpretations after an independent reading of the relevant documents, thereby encouraging the identification of continuities and discontinuities in the document batch. We sought to actively disconfirm interpretations and note that we were unable to do so.

Finally, our study was limited to Coca-Cola, but it is highly likely that other multinational food corporations also seek to exert influence on academic nutrition conferences and events. Future research is needed to extend this study to investigate potential food industry influence among different companies and at academic conferences.

### Implications for research and policy

Notwithstanding these caveats, our findings have significance for public health policy and especially conference management. Our analysis demonstrates that the prominence of Coca-Cola as a funding source has been obscured in several leading conferences. This appears to be by design; individuals involved received guidance specifically not to mention Coca-Cola. We therefore observe that Coca-Cola-academic relationships, both financial and non-financial, are often not visible to attendees or the public. On multiple occasions, Coca-Cola supported and selected conference speakers without being listed as an official sponsor, and without researchers declaring how their participation was funded. Financing was difficult to track, and we identify instances where it was funnelled via affiliated non-profit organisations. For participants at conferences, it is impossible to differentiate information produced via industry ties.

Our research highlights the use of Coca-Cola’s ‘soft power’ to attempt to set the public health agenda on obesity and nutrition^(51)^. By sponsoring conferences, Coca-Cola can directly shape what is included and not included in conference proceedings. This is of tremendous value to the company. It can promote its own favoured solutions to obesity and public health nutrition more widely. It can also shape the narrative about what constitutes appropriate interventions. Participating in mainstream public health events can lend credibility to Coca-Cola itself and its messaging and create a platform for further media engagement. Coca-Cola often conditioned payments to researchers on engaging in media events that it organised in connection with the conference.

With a rapid return to events and conferences in the post-pandemic period, the implications are clear. There should be robust financial and conflict-of-interest disclosures for public health conferences, not only for the conference organisers, but also for speakers. Policymakers and institutions should insist upon researchers including declarations on their presentations, websites and research outputs that indicate potential influences on their work and all funding received not just across the project being discussed but more widely. Only by making transparent the full financial relationships between companies, researchers and institutions, the public, researchers and policymakers will be better able to fully understand influence and to interpret any bias in research outputs. Requiring researchers and institutions to highlight funding and relationships in outputs and in press activity is a critical step towards transparency.

## Appendix I List of FOI document requests, sorted by request date and corresponding time frame

**Table:**

Name	Request date	Request Time frame	Notes
DianaThomas_Emails1	19/07/2018	1/1/12–7/31/16	
DianaThomas_Emails2	15/08/2018	1/1/12–7/31/16	
Greg Hand 1	04/08/2015	1/1/13-present	
Greg Hand 2018 Part 1	05/10/2017	8/1/14-present	A portion was for 8/1/15-present
Joanne Slavin	22/12/2016	1/1/14-present	Received: 1/1/14–1/1/16
PBRC Church + Katzmarzyk 1	19/09/2016	1/1/12-present	
PBRC Church + Katzmarzyk 2	06/10/2017	9/1/16-present	
PBRC Church + Katzmarzyk 3	09/04/2018	1/1/12-present	
PBRC Claude Bouchard	22/09/2017	1/1/12-present	
PBRC Steven Heymsfield Coke	25/07/2018	1/1/13-present	
Alan Boobis – Coke	04/04/2017	1/1/12-present	
Steve Blair	14/08/2015	1/1/13-present	
James Hill 1	15/07/2016	1/1/13-present	
James Hill 2	03/02/2017	1/1/12-present	
James Hill 3	18/10/2017	1/1/13-present	Portions from 1/1/16 and 7/1/16 – present
John Peters 1	17/07/2015	1/1/12-present	
Peters Malaspina	21/09/2017	1/1/11-present	
Russ Pate Part 1	12/10/2017	1/1/13-present	
Adam Drewnowski 1	17/07/2015	1/1/12-present	
Adam Drewnowski 2	20/01/2016	1/1/12-present	Different entities from Coca-Cola1
Adam Drewnowski 3	25/09/2017	1/1/16-present	

## Appendix II I List of scientific events identified in the dataset

Influence by Coca-Cola is classified into three categories:

- A = Direct financial contributions to conference organisers
- B = Indirect financial support through non-profit organisations
- C = Direct financial contributions to conference speakers

**Table:**

Full name	Date	Type of event	Host organisation	Type of influence	Topic
National Physical Activity Plan Congress 2015	Feb-15	Conference	National Physical Activity Plan Alliance	A	Physical activity
Scientific and Coordinating Committee for Health Measurement Meeting	Jan-13	Private meeting	Westat	Not identified	General/Unclassifiable
Sedentary Behavior: Identifying Research Priorities	Jun-13	Workshop	National Health, Lung and Blood Institute and National Institute of Aging	Not identified	Physical activity
Southeastern Conference Symposium 2014	Sep-14	Symposium	SECU	A	Obesity
ILSI North-Andean Conference	Sep-14	Forum	ILSI North-Andean and the Colombian Employer Association (ANDI)	B	Soda tax
Together, We Can Unite for Healthier Kids	Jun-16	Private meeting	Nestlé	Not identified	Obesity
Active Healthy Living Stakeholder Convening	Nov-13	Private meeting	The Coca-Cola Company and Business for Social Responsibility	A	Physical activity
Forum on Healthy Behavior Change 2013	May-13	Forum	Kaiser Permanente and American Heart Association	Not identified	Obesity
European College of Sport Sciences 20th Annual Congress 2015	Jun-15	Conference	European College of Sport Sciences	B	Physical activity
9th International Conference on Diet and Activity Methods 2015	Sep-15	Conference	International Society of Behavioral Nutrition and Physical Activity	B	Obesity
Early Career Course	Jun-15	Workshop	International Society for Physical Activity and Health	Not identified	Physical activity
The Obesity Society Annual Meeting 2013	Nov-13	Conference	The Obesity Society and the American Society for Metabolic and Bariatric Surgery	B	Obesity
Excellence in Pediatrics Conference	Dec-15	Conference	Excellence in Pediatrics	B	Pediatrics
Nutrition Society of Australia Annual Conference 2014	Nov-14	Conference	Nutrition Society of Australia	A	Nutrition
The Obesity Society Annual Meeting 2015	Nov-15	Conference	The Obesity Society and the American Society for Metabolic and Bariatric Surgery	B	Obesity
The Obesity Society Annual Meeting 2014	Nov-14	Conference	The Obesity Society and the American Society for Metabolic and Bariatric Surgery	B	Obesity
ANZOS/ALMA Annual Scientific Meeting	Oct-14	Conference	The Australia and New Zealand Obesity Society	C	Obesity
International Society of Behavioral Nutrition And Physical Activity Conference 2014 San Diego	May-14	Conference	International Society of Behavioral Nutrition and Physical Activity	B	Obesity
AAP Experience National Conference and Exhibition 2014	Oct-14	Conference	American Academy of Pediatrics	Not identified	Pediatrics
EPODE International Network Meeting 2012	Jun-12	Conference	Global Obesity Forum	B	Obesity
IUNS International Congress of Nutrition 2013	Sep-13	Conference	International Union of Nutritional Sciences	A	Nutrition
IUNS International Congress of Nutrition 2009	Sep-09	Conference	International Union of Nutritional Sciences	Not identified	Nutrition
South Africa Nutrition Congress 2014	Sep-14	Conference	Nutrition Society of South Africa and Association for Dietetics in South Africa	C	Nutrition
International Congress on Physical Activity and Public Health 2012	Oct-12	Conference	Sports Medicine Australia	A	Physical activity
ACSM National Strategic Summit 2012	Sep-12	Conference	American College of Sports Medicine	B	Physical activity
ACSM Annual Meeting 2015	Sep-15	Conference	American College of Sports Medicine and Exercise is Medicine	B	Physical activity
ACSM Annual Meeting 2013	Jul-13	Conference	American College of Sports Medicine and Exercise is Medicine	B	Physical activity
ACSM Annual Meeting 2014	May-14	Conference	American College of Sports Medicine and Exercise is Medicine	B	Physical activity
ACSM Annual Meeting 2016, World Congress on the Basic Science of Energy Balance	May-16	Conference	American College of Sports Medicine and Exercise is Medicine	Not identified	Physical activity
CONNECT Physiotherapy Conference 2015	Oct-15	Conference	Australian Physiotherapy Association	C	Physical activity
Balancing the Debate Symposium 2012	Jan-12	Symposium	The Coca-Cola Company	A	Collaboration with industry
Canadian Obesity Summit	Apr-15	Conference	The Canadian Obesity Network	C	Obesity
Energy Balance session	Jan-15	Private meeting	The Coca-Cola Company	A	Obesity
Energy Transition and public health	May-14	Conference	The Royal Society of Medicine	C	Obesity
Exercise is Medicine conference 2013	Jun-13	Conference	Exercise is Medicine	B	Physical activity
Exercise is Medicine at the Royal Society of Medicine 2015	Jun-15	Conference	The Royal Society of Medicine	C	Physical activity
Exercise is Medicine conference 2014	Jun-14	Conference	Exercise is Medicine	B	Physical activity
Cardiovascular Seminar	Nov-15	Seminar	American Heart Association	C	Cardiology
US-Russia Scientific Forum 2011	Nov-11	Conference	National Institute for Health	A	Collaboration with industry
Carnegie Research Seminar Series	Jan-16	Seminar	Leeds University	C	General/Unclassifiable
Gold Coast Sports Physicians Conference	Feb-16	Conference	Australasian College of Sport and Exercise Physicians	Not identified	Physical activity
2nd Global Congress for Consensus in Pediatrics and Child Health	May-12	Conference	Consensus in Pediatrics	A	Pediatrics
Fitness over Fatness discussions	Aug-15	Webinar	Eat To Perform	C	Obesity
12th European Nutrition Congress	Oct-15	Conference	Federation of European Neuroscience Societies	C	Nutrition
European College of Sport Sciences 19th Annual Congress 2014	Jul-14	Conference	European College of Sport Sciences	A	Physical activity
33rd International Symposium of Diabetes and Nutrition 2015	Jun-15	Symposium	Diabetes and Nutrition Study Group	A	Diabetes
3rd International Weight Stigma Conference	Sep-15	Conference	Stigma Conferences	Not identified	Obesity
3rd World Congress on Public Health Nutrition 2014	Nov-14	Conference	Nutrición Sin Fronteras and more…	A	Nutrition
Exercise is Medicine China 2014	Nov-14	Conference	ILSI Focal Point China	B	Physical activity
The future of EIM	Apr-15	Symposium	Exercise is Medicine	B	Physical activity
Centre for Evidence-Based Medicine Conference	Jun-16	Conference	University of Oxford	Not identified	General/Unclassifiable
Scientific Sessions	Nov-15	Conference	American Heart Association	Not identified	Cardiology
ILSI meeting, China	Dec-11	Private meeting	International Life Sciences Institute (ILSI) China	B	General/Unclassifiable
China National Healthy Lifestyle Conference	Aug-15	Conference	CDC China	B	Obesity
Energy Balance workshop	Sep-14	Private meeting	International Life Sciences Institute (ILSI)	B	Obesity
World Congress of Cardiology Science	Apr-12	Conference	World Heart Federation and European Society of Cardiology	Not identified	Cardiology
Energy Balance forum	Dec-14	Forum	Coca-Cola Amatil	A	Obesity
The 39th Annual International Conference on Global Health	Jul-12	Conference	Global Health Council	C	Obesity
International Conference on Science, Education and Medicine	Jul-12	Conference	International Council of Sport Science and Physical Education	A	Physical activity
Exercise is Medicine seminar	Dec-15	Seminar	Exercise is Medicine	B	Physical activity
Physical Activity and Public Health Conference	Sep-12	Lectures	University of South Carolina Prevention Research Center and CDC	Not identified	Physical activity
International Congress on Physical Activity and Public Health 2014	Apr-14	Conference	International Council of Sport Science and Physical Education	C	Physical activity
International Society of Behavioral Nutrition And Physical Activity Conference 2015 Edinburgh	Jun-15	Conference	International Society of Behavioral Nutrition and Physical Activity	C	Obesity
Exercise Towards Health	Nov-14	Lectures	American Heart Association	C	Physical activity
Israeli Physical Therapy Conference	May-14	Conference	Israeli Physiotherapy Society	Not identified	Physical activity
Harvard Physical Activity Course	Feb-15	Lectures	Harvard University	Not identified	Physical activity
University of Queensland lectures	Oct-15	Lectures	University of Queensland	Not identified	General/Unclassifiable
Physical Inactivity Talk at Grand Forks	Sep-14	Lectures	University of North-Dakota	Not identified	Physical activity
Australian-Pacific Obesity Conference 2014	Oct-14	Conference	Unknown	Not identified	Obesity
Spanish Federation of Nutrition Societies Meeting	unknown-14	Private meeting	Spanish Federation of Nutrition Societies	C	Collaboration with industry
Brown Bottle Series	May-15	Lectures	University of South Carolina	B	General/Unclassifiable
Ironman Sports Medicine Conference	Oct-11	Conference	Ironman	Not identified	Physical activity
International Conference on Obesity	Aug-06	Conference	World Obesity Federation	Not identified	Obesity
World Aquatic Health Conference (WAHC) 2015	Oct-15	Conference	National Swimming Pool Foundation	Not identified	Physical activity
Sedentary Behavior and Health Conference	Sep-15	Conference	University of Illinois at Urbana-Champaign	Not identified	Physical activity
San Diego State University’s 100-year anniversary	Apr-15	Lectures	San Diego State University	C	General/Unclassifiable
Health, Kinesiology and Sport management in the SEC	Apr-15	Lectures	SECU	C	Physical activity
AIESEP 2015	Jul-15	Conference	International Association for Physical Education in Higher Education	C	Physical activity
Diploma in Sports Nutrition	Apr-15	Lectures	International Olympic Committee	C	Physical activity
Nutritional Biology Seminar Series	May-15	Lectures	UC Davis	C	Nutrition
16th Biennial Congress	Oct-15	Conference	South African Sports Medicine Association (SASMA)	C	Physical activity
AND Conference	Apr-12	Conference	Academy of Nutrition and Dietetics (AND)	B	Nutrition
6th Brazilian Congress of Integrated Nutrition	Jun-15	Conference	Ganepão	B	Nutrition
ESLM Webinar	May-15	Webinar	European Society for Lifestyle Medicine	A	Obesity
Food in the New Era: The International Conference of the Israeli Food Industry	Jun-15	Conference	Israeli Ministry of Economy and the Israeli Chamber of Commerce	C	Collaboration with industry
EIM Israel	Unknown-14	Conference	Exercise is Medicine	B	Physical activity
Medical School Grand Rounds	Feb-15	Lectures	Florida State University	C	General/Unclassifiable
Cardiovascular Grand Rounds	Oct-13	Lectures	Harvard University	C	Cardiology
Buena Vista: Expanding Horizons in Behavioral Medicine	Mar-17	Symposium	Society of Behavioral Medicine	Not identified	General/Unclassifiable
NAK Annual Meeting 2015	Sep-15	Conference	US National Academy of Kinesiology	Not identified	Physical activity
22nd Annual Congress	Jul-17	Conference	European College of Sport Sciences	Not identified	Physical activity
Symposium 2011	Jan-11	Symposium	The Coca-Cola Company	A	Coca-Cola
23nd Puijo Symposium	Jun-14	Symposium	Kuopio Research Institute of Exercise Medicine	C	Physical activity
Ponte al 100, Balance Energético	Aug-14	Conference	Ponte al 100	A	Obesity
Sports Surgery Clinic	Unknown-14	Conference	Sports Surgery Clinic	Not identified	Physical activity
Childhood Obesity & Public Health Conference	Dec-14	Conference	Pennington Biomedical Research Center	C	Obesity
Coke Conference	Apr-12	Conference	The Coca-Cola Company	A	Coca-Cola
EAS Congress	Mar-15	Conference	European Atherosclerosis Association	C	Cardiology
Northwest ACSM	Feb-15	Lectures	American College of Sports Medicine	C	Physical activity
4th World Conference on Positive Psychology	Jun-15	Conference	International Psychology Association	Not identified	General/Unclassifiable
West Virginia University College of Physical Activity and Sport Sciences	Oct-16	Lectures	West Virginia University	Not identified	Physical activity
Annual meeting 2012	Sep-12	Conference	Cardiac Society of Australia and New Zealand	Not identified	Cardiology
NAK Annual Meeting 2012	Oct-12	Conference	US National Academy of Kinesiology	Not identified	Physical activity
Dilemmas in Cardiovascular Disease Meeting	Oct-12	Seminar	Unknown	Not identified	Cardiology
AMTA Meeting	Oct-12	Conference	America Massage Therapy Association	Not identified	General/Unclassifiable
Get Moving Tasmania Conference	Nov-12	Conference	Tasmanian Government	Not identified	Physical activity
CLARITY Meeting	Nov-12	Private meeting	Unknown	Not identified	General/Unclassifiable
ICAA Conference 2012	May-12	Conference	International Council of Active Aging	Not identified	Physical activity
Physical Activity Conference	Dec-12	Conference	National Institute for Health	Not identified	Physical activity
ILSI North America Meeting	Jan-13	Conference	International Life Sciences Institute (ILSI)	B	General/Unclassifiable
Energy Balance expert discussion meeting	Jan-13	Symposium	International Life Sciences Institute (ILSI)	B	Obesity
ƒInternational Consensus Conference on Energy Balance	Dec-14	Conference	International Life Sciences Institute (ILSI)	B	Obesity
ASPC/AHA Annual Meeting	May-13	Conference	Association of Surgeons in Primary Care and American Heart Association	Not identified	Cardiology
Coca-Cola Conference Aruba	Mar-13	Conference	The Coca-Cola Company	A	Coca-Cola
EASO Meeting	May-13	Conference	The European Congress of Obesity	C	Obesity
Cardiff Exercise Medicine Symposium	Jun-13	Symposium	British Association of Sport & Exercise Medicine	Not identified	Physical activity
NBNA Conference 2012	Jul-12	Conference	National Black Nurses Association	C	General/Unclassifiable
NACDS Total Store Expo	Aug-13	Conference	National Association of Chain Drug Stores	A	Collaboration with industry
Distinguished Lecture Series	Apr-15	Lectures	Texas A&M University	Not identified	General/Unclassifiable
Metro South Conference	May-15	Conference	Unknown	Not identified	General/Unclassifiable
Harvard Medical School Lecture	Dec-15	Lectures	Harvard University	Not identified	General/Unclassifiable
ACC Conference 2015	Mar-15	Conference	American College of Cardiology	Not identified	Cardiology
Interview on sweeteners	Nov-12	Interview	Coca-Cola	A	Sweeteners
Media Medics Roundtable	Nov-12	Private meeting	Media Medics	C	General/Unclassifiable
13th Panhellenic Congress on Diabetes	Mar-13	Conference	Hellenic Diabetes Association	C	Diabetes
ESAC meeting 2013	Apr-13	Private meeting	European Scientific Advisory Council	C	Sweeteners
ISA Conference 2014	Apr-14	Conference	International Sweeteners Association	C	Sweeteners
EUROTOX 2014	Sep-14	Conference	European Tox Forum	C	Sweeteners
Thirst for Knowledge Panel Discussion	Nov-13	Panel Discussion	The Coca-Cola Company	A	Sweeteners
Nutrition and Health Live	Nov-13	Webinar	The Coca-Cola Company	A	Sweeteners
Risk Assessment Conference	Jun-14	Conference	International Life Sciences Institute (ILSI) South Africa	B	Sweeteners
Conference London 2013	Mar-13	Conference	Imperial College London	C	General/Unclassifiable
SRA Global Forum	Apr-13	Conference	The Coca-Cola Company	A	Coca-Cola
National Soda Summit	Jun-12	Conference	Center for Science in the Public Interest	Not identified	Nutrition
Obesity Week 2013 symposium	Nov-13	Symposium	The Obesity Society	C	Obesity
ECO 2012	May-12	Conference	The European Congress of Obesity	C	Obesity
ECO 2011	May-11	Conference	The European Congress of Obesity	Not identified	Obesity
Directors Meeting	Jul-14	Private meeting	National Cancer Institute	Not identified	General/Unclassifiable
National Conference and Exhibition Symposium	Oct-15	Symposium	American Academy of Pediatrics	Not identified	Pediatrics
International Conference on Pediatrics (ICP) 2013	Aug-13	Conference	WHO	C	Pediatrics
CPEStt-EMG Annual Conference 2013	Jun-13	Conference	Physical Education and Sport Think-Tank and Exercise Medicine Ghana	C	Physical activity
Colombian Congress on Pediatrics 2013	Jun-13	Conference	Latino American Association of Pediatrics	C	Pediatrics
Global Summit on Physical Activity of Children	May-14	Conference	International Council of Sport Science and Physical Education	C	Physical activity
International Congress on Obesity 2014	Mar-14	Conference	World Obesity Federation	C	Obesity
American Public Health Association Annual Meeting	Nov-14	Conference	American Public Health Association	C	General/Unclassifiable
International Symposium on Minority Health & Health Disparities	Dec-14	Conference	National Institute on Minority Health & Health Disparities	C	General/Unclassifiable
CSEP Annual General Meeting	Oct-14	Conference	Canadian Society for Exercise Physiology	C	Physical activity
AHK Canada Global Summit	May-14	Conference	Active Healthy Kids Canada	C	Physical activity
AHL Research Conference	Feb-15	Conference	Active Living Research	Not identified	Physical activity
25th Congress of the Nutrition Society of South Africa	Sep-14	Conference	Association for Dietetics	C	Nutrition
Conference on Physical Activity and Sports for Health and Development in Africa	Mar-14	Conference	University of Ghana	C	Physical activity
Epidemiology and Prevention/Nutrition, Physical Activity and Metabolism Scientific Sessions	Mar-14	Conference	American Heart Association	Not identified	Cardiology
Conference in Lebanon 2014	Oct-14	Conference	American University of Beirut	C	Obesity
ICPAPH 2016	Nov-16	Conference	International society for Physical Activity and Health	Not identified	Physical activity
MENA AHL Summit	Dec-14	Conference	Active Healthy Living, Excellence in Pediatrics	B	Pediatrics
Hydration Deep Dive Day	Unknown-15	Symposium	The Coca-Cola Company	A	Nutrition
World Diabetes Congress	Nov-15	Conference	The International Diabetes Federation	C	Diabetes
LIFE Ancillary Study Symposium	Mar-12	Symposium	Florida State University	C	General/Unclassifiable
ACSM Annual Meeting 2017	Jun-17	Conference	American College of Sports Medicine	Not identified	Physical activity
ACSM Annual Meeting 2018	May-18	Conference	American College of Sports Medicine	Not identified	Physical activity
Obesity Week 2015	Nov-15	Conference	The Obesity Society and the American Society for Metabolic and Bariatric Surgery	C	Obesity
Obesity Week 2016	Nov-16	Conference	The Obesity Society and the American Society for Metabolic and Bariatric Surgery	Not identified	Obesity
Obesity Week 2017	Oct-17	Conference	The Obesity Society and the American Society for Metabolic and Bariatric Surgery	Not identified	Obesity
Experimental Biology (EB) 2017	Apr-17	Conference	American Physiological Society (APS)	Not identified	Physical activity
Experimental Biology (EB) 2015	Mar-15	Conference	American Physiological Society (APS)	Not identified	Physical activity
Experimental Biology (EB) 2012	Apr-12	Conference	American Physiological Society (APS)	Not identified	Physical activity
Genomics, Genetics and Exercise Biology: A Celebratory Symposium	May-15	Symposium	International Federation of Sports Medicine (FIMS)	Not identified	Physical activity
Molecular Transducers Webinar	Unknown15	Webinar	Molecular Transducers of Physical Activity Consortium	Not identified	Physical activity
International Roundtable on the Genetic Regulation of Physical Activity (IR-GRPA)	Mar-17	Panel Discussion	American College of Sports Medicine	B	Physical activity
Basic Science Sessions	Nov-15	Symposium	The Obesity Society		Obesity
A Civil Society Event on Action in Climate Change and Health	Sep-14	Conference	American College of Sports Medicine	B	General/Unclassifiable
AND Food And Nutrition Conference and Expo 2012	Oct-12	Conference	Academy of Nutrition and Dietetics (AND)	B	Nutrition
ILSI North America Second International Conference on Hydration and Health	Nov-11	Conference	International Life Sciences Institute (ILSI)	B	Nutrition
ILSI North America Energy Balance Workshop	Dec-14	Workshop	International Life Sciences Institute (ILSI)	B	Obesity
ILSI Europe 30th Anniversary Annual Symposium	Mar-16	Symposium	International Life Sciences Institute (ILSI)	B	General/Unclassifiable
ILSI Europe 6th International Symposium on Food Packaging	Nov-16	Symposium	International Life Sciences Institute (ILSI) Europe	B	Collaboration with industry
ILSI North America Scientific Session	Jan-12	Symposium	International Life Sciences Institute (ILSI)	B	General/Unclassifiable
ILSI North America Annual Meeting	Sep-17	Conference	International Life Sciences Institute (ILSI)	B	General/Unclassifiable
12th China Nutrition Science Congress	May-14	Conference	Chinese Nutrition Society	Not identified	Nutrition
International Conference on the Role of Diet, Physical Activity & Lifestyle in Promoting Health	Nov-15	Conference	International Life Sciences Institute (ILSI) India	B	Obesity
Healthy Weight, Active Lifestyles Communications Forum	May-12	Private meeting	International Food Information Council (IFIC)	Not identified	Obesity
Latin American and Caribbean Summit on Prevention of Obesity and NCD	Oct-11	Conference	Pan American Health Organization	Not identified	Obesity
ILSI Brasil Carbohydrates Symposium	Dec-11	Symposium	International Life Sciences Institute (ILSI) Brasil	B	Nutrition
Nutrition News Forecast 2014 Session	Unknown-14	Symposium	Academy of Nutrition and Dietetics (AND)	B	Nutrition
Nutrition News Forecast 2015 Session	Apr-15	Symposium	Academy of Nutrition and Dietetics (AND)	B	Nutrition
Communicating the Science of Physical Activity and Health	Dec-12	Panel Discussion	American College of Sports Medicine and the National Press Club	B	Physical activity
Sweetness Symposium	Oct-14	Symposium	European Federation of the Associations of Dietitians (EFAD)	C	Sweeteners
The Interplay between Environmental Exposures and Obesity	Mar-15	Workshop	National Institute of Environmental Health Sciences	Not identified	Obesity
PCNA Webinar	Sep-12	Webinar	Preventive Cardiovascular Nurses Association	C	Cardiology
ADA Scientific Sessions	Jun-14	Symposium	American Diabetes Association	Not identified	Diabetes
10th Symposium on Body Composition	Jun-14	Symposium	University of Lisbon	A	General/Unclassifiable
ILSI GC Annual Meeting	Jan-17	Private meeting	International Life Sciences Institute (ILSI)	B	General/Unclassifiable
ILSI Global Annual Meeting	Jan-18	Conference	International Life Sciences Institute (ILSI)	B	General/Unclassifiable
Annual Meeting of the Chinese Beverage Industry	Dec-14	Private meeting	Unknown	Not identified	Collaboration with industry
EBAL Tech Summit	Sep-16	Conference	International Life Sciences Institute (ILSI)	B	Obesity
4th Annual Conference Exercise is Medicine for the Brain	May-15	Conference	Exercise is Medicine	B	Physical activity
West Virginia Physical Activity Symposium	Mar-15	Symposium	West Virginia Physical Activity Network	Not identified	Physical activity
Obesity Boot Camp	Late-2014	Lectures	Unknown	Not identified	Obesity
EASD Meeting	Sep-14	Private meeting	European Association for the Study of Diabetes	Not identified	Diabetes
Seminar GGNB	Aug-14	Seminar	UC Davis	Not identified	General/Unclassifiable
ACSM Symposium 2018	Unknown-18	Symposium	American College of Sports Medicine	Not identified	Physical activity
EAC Global Summit	Jun-17	Conference	Consumer Goods Forum	B	Collaboration with industry
Vision 2028 Summit	Aug-17	Conference	American Society for Nutrition	Not identified	Nutrition
ILSI 2017 Board meeting	Unknown-17	Private meeting	International Life Sciences Institute (ILSI)	B	General/Unclassifiable
Sustainable Retail Summit	Oct-17	Conference	Consumer Goods Forum	B	Collaboration with industry
IFIC Healthy Weight, Active Lifestyles Communications Forum	May-14	Forum	International Food Information Council (IFIC)	C	Obesity
ILSI 2014 Annual Meeting	Jan-14	Conference	International Life Sciences Institute (ILSI)	B	General/Unclassifiable
ILSI 2015 Annual Meeting	Jan-14	Conference	International Life Sciences Institute (ILSI)	B	General/Unclassifiable
Annual Meeting for Health and Wellness Journalists	Apr-13	Conference	National Press Foundation and UC Denver	B	General/Unclassifiable
Obesity Issues 2013	May-13	Conference	National Press foundation and Anschutz Health and Wellness Center	B	Obesity
Obesity Issues 2014	Jun-14	Conference	National Press foundation and Anschutz Health and Wellness Center	B	Obesity
Habit Breaking Workshop	Jul-15	Workshop	Unknown	Not identified	General/Unclassifiable
Conference on Obesity Control and Prevention	Oct-15	Conference	International Life Sciences Institute (ILSI) China	B	Obesity
ILSI North America Food, Nutrition & Safety Program (FNSP)	Jul-14	Conference	International Life Sciences Institute (ILSI)	B	Nutrition
Neurocognition: the Food and Brain Connection	Apr-14	Symposium	American Society for Nutrition	Not identified	Nutrition
Food and Nutrition Conference and Exhibition (FNCE)	Sep-14	Conference	Academy of Nutrition and Dietetics (AND)	B	Nutrition
High intensity sweeteners, health benefits and application in food and beverage industry	Oct-14	Symposium	International Life Sciences Institute (ILSI) Mexico	B	Sweeteners
ASN Independent meeting	Unknown-14	Private meeting	American Society for Nutrition	Not identified	Nutrition
Networking Breakfast	Oct-15	Private meeting	Calorie Control Council	B	Collaboration with industry
The CGF Health and Wellness Pillar Steering Committee Meeting	Sep-15	Private meeting	Consumer Goods Forum	B	Collaboration with industry
NAM Annual Meeting	Jan-16	Conference	National Academy of Medicine	Not identifiedGeneral/Unclassifiable	
ILSI North America Future Leader Reception	Apr-16	Private meeting	International Life Sciences Institute (ILSI)	B	General/Unclassifiable
ILSI North America Assembly of Members	Jan-16	Conference	International Life Sciences Institute (ILSI)	B	General/Unclassifiable
4th Nutrition and Healthy Lifestyles Summit	May-17	Conference	Sabri Ulker Food Research Foundation	C	Obesity
Endocrine Grand Rounds	Dec-16	Lectures	Vanderbilt University	Not identified	General/Unclassifiable
CCG Annual Meeting 2016	Nov-16	Conference	Calorie Control Council	B	Obesity
ILSI Technology Summit 2016	Late-2016	Conference	International Life Sciences Institute (ILSI)	B	General/Unclassifiable
Art and Science of Health Promotion Conference	Mar-17	Conference	American College of Lifestyle Medicine	Not identified	General/Unclassifiable
Micronutrients Symposium	Jul-15	Symposium	International Life Sciences Institute (ILSI) Southeast Asia	B	Nutrition
COIN Summit	Aug-15	Conference	Colorado Innovation Network	Not identified	General/Unclassifiable
33rd ISDN International Symposium on Diabetes and Nutrition	Jun-15	Symposium	Interdisciplinary Symposium on Decision Neuroscience	Not identified	Diabetes
Stakeholder Conference 2015	May-15	Private meeting	European Food Information Council	C	Collaboration with industry
Asian Congress of Nutrition	May-15	Conference	International Life Sciences Institute (ILSI) Southeast Asia	B	Nutrition
FDA Roundtable Discussion 2015	Unknown-15	Private meeting	Food and Drug Administration	C	Collaboration with industry
Third Pan American Conference on Obesity	Jun-14	Conference	PACO	A	Obesity
Nutrition Theater	Oct-14	Lectures	American Academy of Family Physicians	B	Nutrition
Massey University Symposium	Dec-14	Symposium	Massey University	C	Obesity
Annual meeting of the Chinese beverage industry	Dec-14	Conference	Unknown	C	Collaboration with industry

## Appendix III List of events where Coca-Cola funded conference organisers

**Table:**

Name	Type of support	Amount of funding	Also funded conference speakers
National Physical Activity Plan Congress 2015	Keynote sponsor	$30 000	Not identified
Southeastern Conference Symposium 2014	Official sponsor	$10 000	Not identified
Active Healthy Living Stakeholder Convening	Hosting event	Unknown	Not identified
Nutrition Society of Australia Annual Conference 2014	Official sponsor	Unknown	Yes
IUNS International Congress of Nutrition 2013	Official publication sponsor	$15 000	Yes
International Congress on Physical Activity and Public Health 2012	Official sponsor	$100 000	Yes
Balancing the Debate Symposium 2012	Hosting event	Unknown	Not identified
Energy Balance session	Hosting event	Unknown	Not identified
US-Russia Scientific Forum 2011	Letting two senior Coca-Cola employees participate	Unknown	Not identified
2nd Global Congress for Consensus in Pediatrics and Child Health	Symposium sponsor	Unknown	Not identified
European College of Sport Sciences 19th Annual Congress 2014	Official sponsor	$30 000	Yes
33rd International Symposium of Diabetes and Nutrition 2015	Official sponsor	Unknown	
3rd World Congress on Public Health Nutrition 2014	Official sponsor	Unknown	Yes
Energy Balance forum	Hosting event	Unknown	
International Conference on Science, Education and Medicine	Official sponsor	Unknown	Yes
ESLM Webinar	Sponsor	Unknown	Not identified
Symposium 2011	Hosting event	Unknown	Not identified
Ponte al 100, Balance Energético	Official sponsor	Unknown	Not identified
Coke Conference	Hosting event	Unknown	Not identified
Coca-Cola Conference Aruba	Hosting event	Unknown	Yes
NACDS Total Store Expo	Official sponsor	$25 000	Yes
Interview on sweeteners	Hosting event	Unknown	Not identified
Thirst for Knowledge Panel Discussion	Hosting event	Unknown	Not identified
Nutrition and Health Live	Hosting event	Unknown	Not identified
SRA Global Forum	Hosting event	Unknown	Not identified
Hydration Deep Dive Day	Hosting event	Unknown	Not identified
10th Symposium on Body Composition	Symposium sponsor	Unknown	Yes
Pan American Conference on Obesity	Coca-Cola Chief Scientific Officer a plenary speaker	Unknown	Not identified
